# Improved understanding of biofilm development by *Piscirickettsia salmonis* reveals potential risks for the persistence and dissemination of piscirickettsiosis

**DOI:** 10.1038/s41598-020-68990-4

**Published:** 2020-07-22

**Authors:** Héctor A. Levipan, Rute Irgang, Alejandro Yáñez, Ruben Avendaño-Herrera

**Affiliations:** 1 Departamento de Biología, Facultad de Ciencias Naturales y Exactas, Universidad de Playa Ancha, Valparaiso, Chile; 2 Laboratorio de Patología de Organismos Acuáticos y Biotecnología Acuícola, Facultad de Ciencias de la Vida, Universidad Andrés Bello, Viña del Mar, Chile; 3 Interdisciplinary Center for Aquaculture Research (INCAR), Universidad Andrés Bello, Viña del Mar, Chile; 4 Facultad de Ciencias, Universidad Austral de Chile, Valdivia, Chile; 5 Centro de Investigación Marina Quintay (CIMARQ), Universidad Andrés Bello, Quintay, Chile

**Keywords:** Biofilms, Pathogens

## Abstract

*Piscirickettsia salmonis* is the causative agent of piscirickettsiosis, a disease with high socio-economic impacts for Chilean salmonid aquaculture. The identification of major environmental reservoirs for *P*. *salmonis* has long been ignored. Most microbial life occurs in biofilms, with possible implications in disease outbreaks as pathogen seed banks. Herein, we report on an in vitro analysis of biofilm formation by *P*. *salmonis* Psal-103 (LF-89-like genotype) and Psal-104 (EM-90-like genotype), the aim of which was to gain new insights into the ecological role of biofilms using multiple approaches. The cytotoxic response of the salmon head kidney cell line to *P*. *salmonis* showed interisolate differences, depending on the source of the bacterial inoculum (biofilm or planktonic). Biofilm formation showed a variable-length lag-phase, which was associated with wider fluctuations in biofilm viability. Interisolate differences in the lag phase emerged regardless of the nutritional content of the medium, but both isolates formed mature biofilms from 288 h onwards. Psal-103 biofilms were sensitive to Atlantic salmon skin mucus during early formation, whereas Psal-104 biofilms were more tolerant. The ability of *P*. *salmonis* to form viable and mucus-tolerant biofilms on plastic surfaces in seawater represents a potentially important environmental risk for the persistence and dissemination of piscirickettsiosis.

## Introduction

Piscirickettsiosis, or salmon rickettsial septicemia, is a fish disease caused by the facultative intracellular bacterium *Piscirickettsia salmonis*. First described in relation to the Chilean salmon farming industry^1^, this pathogen has since been reported in North America and Europe^2^. A decade ago, *P*. *salmonis* was considered an obligate intracellular pathogen^3^, but later research achieved growth on artificial cell-free media^4,5^. *P*. *salmonis* can survive outside of hosts for a long time as free-living cells, having been detected around salmon farms by qPCR up to 30 days after cage emptying^6^ or as a biofilm mode-of-growth in a marine broth medium for 15–30 days^7^. Biofilm formation is a multi-step process that involves bacterial attachment to surfaces, microcolony formation, growth-dependent maturation, and cell detachment from mature biofilms to colonize new habitats^8^. One persisting question is if the survival behavior of *P*. *salmonis* as a free-living bacterium is linked with a biofilm lifestyle in marine habitats. Bacterial biofilms are nearly ubiquitous in all major habitats^9^, including some synthetic habitats, such as the “plastisphere” of marine environments^10–12^.

*P*. *salmonis* can adhere to living surfaces, such as salmonid macrophages and ova^13,14^, as well as form biofilms on abiotic substrates, such as glass, polystyrene plastic, and mussel shells^7,15,16^. Biofilms are thought to be involved in piscirickettsiosis outbreaks^13,17^, particularly in association with skin lesions that efficiently transmit disease^18^. Nevertheless, information is generally lacking on biofilms as a critical facet for *P*. *salmonis* overcoming adverse environments. This gap in knowledge was articulated at a recent expert workshop held in Puerto Montt, Chile, i.e. a key location for Chilean aquaculture development. A total of 52 priority questions concerning piscirickettsiosis research were drawn up, with question 19 asking, “*How does P. salmonis interact with surfaces and survive outside of hosts?*”^19^.

Two studies indicate that *P*. *salmonis* biofilms can serve as a means of survival and resistance to environmental stressors^7, 15^. However, biofilm development by *P*. *salmonis* as a multi-step process is far from being completely understood. The putative role of biofilms in the persistence and transmission of piscirickettsiosis in aquaculture settings is another point lacking clarity. Fish-body mucosal secretions contain a variety of innate immune molecules and, therefore, serve as host-secreted stressors for fish pathogens^20^. Remarkably, there are no studies about the effect of fish skin mucus on the biofilm behavior of *P*. *salmonis*, which would provide deeper insights on the potential protective effect of skin mucus against piscirickettsiosis. Ultimately, the resilient nature of bacterial biofilms to many stressors^21^ makes these cellular collectives a suitable biological model to obtain insights into how *P*. *salmonis* faces host immunological barriers, such as those found in the skin mucus of fish^22,23^.

Herein, we report the results of daily-frequency observations of the phenotypical and physiological features of *P*. *salmonis* Psal-103 and Psal-104 (LF-89-like and EM-90-like genotype representatives, respectively) during in vitro biofilm formation under two nutritionally contrasting conditions, that is, in a nutrient-enriched medium vs. nutrient-poor seawater. *P*. *salmonis* Psal-103 and Psal-104, collected from biofilms as individual cells and/or as aggregates, were cytotoxic for the salmon head kidney (SHK-1) cell line. Furthermore, both isolates formed biofilms tolerant to Atlantic salmon (*Salmo salar*) skin mucus in seawater. General variations in the cytotoxic response to *P*. *salmonis* could be dependent on interisolate differences during the early colonization of surfaces by this bacterium. The formation of *P*. *salmonis* biofilms on plastic surfaces indicates the existence of abiotic reservoirs (here defined as any inert surface able to harbor bacteria using a biofilm lifestyle). Such reservoirs in aquaculture settings likely favor the persistence and dissemination of virulent varieties of *P*. *salmonis* – varieties able to maintain cell viability and that are tolerant to salmon skin mucus under conditions of severe nutrient starvation in seawater.

## Methods

### Bacterial isolates and routine cultures

Chilean isolates of *P. salmonis* are clustered by the prototypes of strains LF‐89^ T^ and EM‐90 in genogroups 1 and 2, respectively^24^. Recently, genogroups 3 (LF‐89^ T^‐like strains) and 4 (EM‐90‐like strains) were identified^25^. In the present study, *P*. *salmonis* Psal-103 and Psal-104 were chosen as being representative of the LF-89-like and EM-90-like genotypes, respectively; this latter choice was based on a sequencing analysis of genomes. The two isolates were isolated in May and August 2012, respectively. For that, kidney samples were collected from sick cage-grown Atlantic salmon kept in the inner sea of Chiloé Island (Los Lagos Region, Chile) during outbreaks of piscirickettsiosis. Isolates were obtained from kidney samples by direct conventional streaking onto AUSTRAL-TSHem agar plates incubated at 18 °C for 4 to 5 days. Initially, each isolate was confirmed as *P*. *salmonis* through standard phenotyping and PCR using a primer pair targeting a fragment of the internal transcribed spacer (ITS) in the prokaryotic ribosomal operon^26^. Afterwards, the two isolates of interest were routinely cultured at 18 °C for 4 to 5 days in a solid AUSTRAL medium or in a liquid AUSTRAL-SRS medium with agitation at 120 rpm^5^. All strains were stored at − 80 °C in Cryobille tubes (AES Laboratory, France). Also, several glycerol-amended stock cultures (10% v/v) were prepared for each strain in AUSTRAL-SRS medium and then aliquoted in 1 mL volumes in cryotube vials for long-term storage at − 80 °C.

### Cytotoxic trials with biofilm and planktonic inocula of *P*. *salmonis*

SHK-1 cells, a macrophage-like cell line derived from *S*. *salar* head kidney, were grown on 24-well microplates (flat-bottomed; SPL Life Sciences Co., Ltd., Pocheon, Korea) at 1 × 10^5^ cells well^−1^ using the Leibovitz’s L-15 medium (HyClone Laboratories Inc., Logan, Utah, USA). This medium was supplemented with 10% fetal bovine serum (Gibco, Invitrogen Laboratories), 6 mM L-glutamine (Gibco), 15 mM HEPES pH 7.3 (Gibco), and 100 μg mL^−1^/100 IU mL^−1^ streptomycin/penicillin (Gibco). SHK-1 cells were incubated at 18 °C and grown at 70–80% confluence. Before infection assays, SHK-1 cells were washed with a phosphate-buffered saline solution (1X PBS, pH 7.0) and then provided with a fresh antibiotic-free medium (2 mL per well) supplemented with 2% fetal bovine serum (Gibco).

Infections were performed with *P*. *salmonis* Psal-103 and Psal-104 obtained from biofilms as individual cells and/or as aggregates, hereafter referred to as biofilm inocula. Planktonic inocula were also assessed for comparison purposes. For this, the Psal-103 and Psal-104 isolates were routinely grown in liquid AUSTRAL broth until achieving a cell concentration of 1.90 ± 0.42 × 10^6^ and 1.69 ± 0.41 × 10^6^ CFU mL^−1^, respectively. Five glass Petri dishes were inoculated with 20 mL of each culture and then statically incubated at 18° C for 288 h to induce biofilm formation. Afterwards, planktonic cell supernatants were withdrawn, and biofilms growing in the bottom of the Petri dishes were washed twice with 10 mL of sterile AUSTRAL broth by gently shaking the plate manually for 5 min. The washing medium was removed, and one of the washed Petri dishes (per isolate) was randomly selected for subsequent staining with a crystal violet (CV) solution (1% w/v, Winkler Chemistry Inc., Santiago, Chile) at room temperature for 10 min. Afterwards, the staining solution was removed, and the CV-stained Petri dishes were washed with abundant sterile milli-Q water until no more dye was released. CV-stained Petri dishes were dried at room temperature for 15 min, and the formation of biofilms was visualized for confirmation purposes under 1000X magnification by optic microscopy (Supplementary Fig. S1). The remaining washed, but unstained, biofilms were scraped off from the bottom of Petri dishes (with cell scrapers) for concentrating biofilm-harvested cells in 5 mL of sterile AUSTRAL broth and obtaining only one biofilm inoculum per isolate. The resulting biofilm inocula of *P*. *salmonis* Psal-103 and Psal-104 were adjusted to 0.91 ± 0.01 McFarland (i.e., 6.55 ± 2.05 × 10^6^ CFU mL^−1^) and 0.94 ± 0.02 McFarland (not quantifiable by plate counting), respectively. Similarly, the optic density of planktonic bacterial suspensions was adjusted to 0.92 ± 0.03 McFarland (i.e., 6.83 ± 0.04 × 10^6^ CFU mL^−1^) and 0.90 ± 0.03 McFarland (not quantifiable by plate counting) to prepare planktonic inocula of *P*. *salmonis* Psal-103 and Psal-104, respectively.

SHK-1 cells were separately infected with biofilm or planktonic inocula by adding 100 µL per well of the McFarland-adjusted inoculum, prepared as described above. Considering the inocula sizes (in CFU mL^−1^) for the biofilm and planktonic suspensions of isolate Psal-103, each experiment was performed using a multiplicity of infection approximately equal to 7. Three independent experiments (or biological replicates) were conducted, with each using triplicate infections at 18 °C for 72 h. During this infection period, aliquots (100 μL) were collected at various hours post-infection (i.e., 0, 6, 12, 24, 48, and 72 hpi) from each well to measure lactate dehydrogenase (LDH)-based cytotoxicity. This was assessed with the Takara Kit (Bio Inc., Otsu, Japan) by spectrophotometric reading at 500 nm (Tecan microplate reader, Infinite 200 PRO, Männedorf, Switzerland). Resulting absorbance measurements were corrected with the background absorbance found in low controls (i.e., non-infected SHK-1 cells). Using the equation provided by the manufacturer of the Takara Kit, measurements were expressed as a percentage of the LDH-based cytotoxicity found in high controls (i.e., SHK-1 cells incubated without bacteria but with 1% Triton X-100).

### Specific biofilm formation index

The two *P*. *salmonis* isolates were routinely grown in AUSTRAL broth (as indicated above) until reaching the exponential growth phase. These cultures were used to inoculate 96-well microplates (flat-bottomed, SPL Life Sciences Co., Ltd.) to determine the specific biofilm formation (SBF) index^27^, which relates sessile growth normalized to planktonic growth. The average inoculum size (determined on AUSTRAL agar plates) was 0.82 ± 0.01 McFarland (i.e., 4.10 ± 3.44 × 10^6^ CFU mL^−1^) and 0.83 ± 0.00 McFarland (i.e., 4.85 ± 3.82 × 10^6^ CFU mL^−1^) for isolates Psal-103 and Psal-104, respectively. Another culture of each isolate was set up to harvest cells in the exponential phase by centrifugation at 4,660 g for 5 min at 4 °C. Afterwards, bacterial pellets of both isolates were washed twice with autoclaved seawater and harvested again by centrifugation. The resulting Psal-103 and Psal-104 pellets were suspended in autoclaved seawater at 0.81 ± 0.04 McFarland (i.e., 4.56 ± 1.59 × 10^6^ CFU mL^−1^) and 0.75 ± 0.03 McFarland (i.e., 4.99 ± 3.07 × 10^6^ CFU mL^−1^), respectively. Three independent experiments were performed by inoculating each well (10 wells per inoculum) with 150 μL of bacterial suspension in AUSTRAL broth or seawater, along with negative controls (i.e., sterile AUSTRAL broth or seawater depending on the inoculum). For each independent experiment, twenty 96-well microplates were inoculated (as described before) and then statically incubated at 18 °C. Afterwards, individual microplates were processed at 24 h intervals over a total period of 480 h to evaluate biofilm formation in nutrient-enriched AUSTRAL broth and nutrient-poor seawater.

After incubation, supernatants from the inoculated and negative-control wells were carefully withdrawn and loaded onto new 96-well microplates to measure absorbance at 620 nm with a Tecan microplate reader (Infinite 200 PRO). Simultaneously, bacteria adhered to the wells of microplates used to evaluate biofilm formation were stained with 180 μL of CV solution (1% w/v) at room temperature for 10 min. The CV solution was eliminated, and the wells were washed with abundant sterile milli-Q water until no more dye was released. Each microplate was placed upside down to dry at room temperature for 15 min. Absolute ethanol (200 μL) was then added to each well for CV solubilization at room temperature for 10 min. The resulting CV solution was homogenized by repeated pipetting before reading the microplates at 590 nm (Infinite 200 PRO). The SBF index was computed as $(B-NC)/G$, where B is the amount of ethanol-solubilized CV that was released from biofilms; NC is the amount of ethanol-solubilized CV that adhered to well surfaces used as negative controls, and G is the absorbance of planktonic bacteria measured at 620 nm from microplate supernatants, as described above.

### Phenotypic and physiological characterization of biofilms

Qualitative and quantitative changes in the surface coverage of biofilms and in the viability of sessile cells, respectively, were analyzed at 18 °C in three independent experiments at 24 h intervals over a total period of 360 h. The two *P*. *salmonis* isolates were routinely grown in AUSTRAL broth (as indicated above), and the average inoculum size was determined on AUSTRAL agar plates (mean ± SD): 0.81 ± 0.02 McFarland (i.e., 7.88 ± 1.87 × 10^6^ CFU mL^−1^) and 0.81 ± 0.03 McFarland (i.e., 1.61 ± 0.74 × 10^6^ CFU mL^−1^) for isolates Psal-103 and Psal-104, respectively. Another culture of each isolate was harvested in the exponential phase by centrifugation at 4,660 g for 5 min at 4 °C. Bacterial pellets of the two isolates were washed twice with autoclaved seawater and harvested again by centrifugation. The resulting Psal-103 and Psal-104 pellets were suspended in sterile seawater (by autoclave) at 0.82 ± 0.03 McFarland (i.e., 9.93 ± 7.84 × 10^6^ CFU mL^−1^) and 0.82 ± 0.02 McFarland (i.e., 1.50 ± 1.21 × 10^6^ CFU mL^−1^), respectively. Afterwards, 96-well microplates were inoculated in triplicate at 24 h intervals with 150 µL of each bacterial suspension in AUSTRAL broth or seawater. This allowed biofilms of different developmental stages (i.e., from the early colonization stage until achieving mature biofilms) to be analyzed on a single microplate. Triplicate 150 µL-aliquots of sterile AUSTRAL broth or seawater were used as negative controls in each experiment assessing biofilm formation under nutrient-enriched or nutrient-poor conditions, respectively.

Prior to the qualitative and quantitative analyses of biofilms formed on 96-well microplates, preliminary experiments were conducted to evaluate the capacity of the LIVE/DEAD BacLight Bacterial Viability Kit (Invitrogen, Molecular Probes, Oregon, USA) to detect physiologically contrasting states of *P*. *salmonis*. Pearson’s correlation coefficients between expected (0: 100, 10: 90, 50: 50, 90:10, and 100: 0) and empiric ratios of live-to-dead cells in bacterial suspensions indicated that the kit was suitable to detect physiologically contrasting states of *P*. *salmonis* Psal-103 (r = 0.9954, *P* < 0.05) and Ps-104 (r = 0.9899, *P* < 0.05). Bacterial suspensions were prepared in a 2.5% NaCl solution per the LIVE/DEAD Kit instruction manual, except that dead cells were fixed in formaldehyde 10% v/v. Empiric live-to-dead bacteria ratios were calculated from bacterial counts obtained with an Olympus BX41 microscope using 1000X magnification (Olympus Corporation, Tokyo, Japan).

Following incubation, the inoculated and negative-control wells of microplates were completely emptied and washed three times with 200 μL of sterile NaCl solution (2.5% w/v). Washed biofilms were stained with the LIVE/DEAD Kit following the manufacturer’s instructions, except that dye salts were dissolved in a NaCl solution (2.5% w/v in sterile milli-Q water) for the staining of *P*. *salmonis* biofilms. The automatic capture of endpoint images (single-point reading) was performed on a Cytation 5 Imaging Multi-Mode Reader (BioTek Instruments Inc., Winooski, VT, USA) using the 20X objective with its correction collar set to 0.5 mm for the usual bottom thickness of 96-well microplates. Images were captured in two fluorescence channels; green (GFP: 469, 525) and acridine orange (AO: 469, 647). Optimal image exposure was achieved using LED intensities of 3 and 5 (GFP and OA channels, respectively), an integration time of 10 and 35 ms (GFP and OA channels, respectively), and a camera gain equal to zero (both channels). The laser autofocus method was used. In this method, the focus position is found using a laser beam that also corrects for any plastic variations. Once the trained position was found, the instrument used the described imaging capture channels and settings to automatically capture the *P*. *salmonis* biofilms for each well. Laser autofocus enables quick and reproducible sample focusing, with the added advantage of having minimal exposure to light. The bottom elevation defined for the used microplate was 3,250 microns. The bottom elevation parameter refers to the distance in microns between the top of the microplate carrier and the bottom of the well where the sample is visible. No additional offset from this setting was required as the laser autofocus system was trained with a reference captured image, and, from this, the system determined the offset to match the desired focusing position if any variation per well was detected.

Furthermore, endpoint fluorescence intensity signals were quantified for each well using the Cytation 5 cell imaging multi-mode reader with a monochromator-based system, with excitation wavelength and bandwidth set to 485/20 nm and emission wavelengths and bandwidths set to 530/20 nm (green emission for live bacteria) and 630/20 nm (red emission for dead or damaged bacteria). The “wavelength switch per well” feature was set to on for sequential signal detection of both emissions before moving to the next well. Reads were performed using top fluorescence optics, a normal read speed (i.e., delay after plate movement of 100 ms), 10 measurements per data point, a height of 7 mm, and an extended dynamic range for automatic gain adjustment. Fluorescence data were corrected by the background fluorescence emitted by negative-control wells and were then expressed as the ratio of the green-to-red fluorescence. This ratio was used as a proxy to evaluate changes in the cell viability of biofilms^28^.

### Collection and characterization of *Salmo salar* skin mucus

A customary procedure for collecting mucus from benzocaine-anesthetized fish (30 mg L^−1^) was used^29^. Healthy *P*. *salmonis*-free adult Atlantic salmon (weighing 850 g) were collected from a fish farm with no history of diseases (Curarrehue Fish Farms, Araucania Region, Chile). All fish were handled by the tail with gloves. Each fish was cleaned through a quick dip into autoclaved seawater to remove any dirt and benzocaine traces. Fish were certified per health regulations as being free of any pathogen described in Chile (e.g., *P*. *salmonis*, infectious salmon anemia virus, infectious pancreatic necrosis virus, *Flavobacterium psychrophilum*, and *Renibacterium salmoninarum*)^30^. Additionally, to ensure that fish were uninfected with *Vibrio ordalii* and *Tenacibaculum dicentrarchi*, samples were subjected to standard microscopic and bacteriological examinations, as well as PCR analysis^31,32^.

Mucus was carefully collected by scraping both sides of the body surface (between the dorsal fin and lateral line) with a sterile stainless-steel spatula. This procedure prevented intestinal contamination from contact with the ventral-anal area. The stainless-steel spatula was also passed from the operculum to the caudal peduncle. The mucus samples were centrifuged for 5 min at 4 °C to remove particulate material, and the supernatant was thoroughly dissolved in sterile milli-Q water (1:1) by repeated pipetting. Resulting mucus solutions were filtered at 0.22 μm by a syringe filter and streaked (10 μL) onto plates containing the AUSTRAL medium, trypticase soy agar supplemented with or without 1% (w/v) NaCl, or Columbia sheep blood agar. The plates were then incubated at 18 °C for 7 days to check for bacterial contamination.

Protein concentrations in mucus samples were determined using a Micro BCA Protein Assay (Pierce, Waltham, MA, USA). Furthermore, mucus samples were examined by sodium dodecyl sulfate–polyacrylamide gel electrophoresis^33^ and silver staining^34^. The lysozyme activity was measured in skin mucus samples by turbidometry at 450 nm^35^ using the Tecan microplate reader (Infinite 200 PRO) and a lysozyme detection kit per the manufacturer’s instructions (Sigma Chemicals Co., St. Louis, MO, USA). This kit uses the bacterium *Micrococcus lysodeikticus* as the source of the substrate (glycosidic linkages between *N*-acetylmuramic acid and *N*-acetylglucosamine in the bacterial cell wall) to measure the lysozyme activity in test samples. Briefly, a suspension of *M. lysodeikticus* (0.25 mg mL^−1^) in PBS (0.004 M, pH 5.8) was incubated at 25 °C for 30 min, and then 190 µL of this suspension was added per well on a 96-well microplate, along with 10 µL of mucus samples adjusted at 100 µg total protein mL^−1^. Blank wells containing 190 µL of the *M*. *lysodeikticus* suspension plus 10 µL of PBS were included per assay. The activity was calculated using the initial rate of reaction, where 1 U of activity was defined as the amount of enzyme that catalyzed a 0.001 min^−1^ drop in absorbance (at 450 nm). One milliliter aliquots of skin-mucus samples (at 100 mg mL^−1^) were stored at − 80 °C until further use.

### Effects of *Salmo salar* skin mucus on biofilm formation

Sterile stock solutions of skin-mucus samples were diluted in autoclaved seawater to 100 µg total protein mL^−1^ (hereafter mucus-amended seawater). This was done to determine the effect of mucus on biofilms formed by *P*. *salmonis* Psal-103 and Psal-104 under nutrient-poor conditions at 18 °C. Briefly, cultures of *P*. *salmonis*, prepared in sterile AUSTRAL broth, were harvested in the exponential phase by centrifugation, as described above. Bacterial pellets were washed twice with autoclaved seawater and harvested again by centrifugation. The resulting Psal-103 and Psal-104 pellets were resuspended in autoclaved seawater (with and without mucus) at 0.81 ± 0.02 McFarland (i.e., 3.12 ± 1.69 × 10^6^ CFU mL^−1^) and 0.84 ± 0.01 McFarland (i.e., 3.58 ± 1.84 × 10^6^ CFU mL^−1^), respectively. To achieve the formation of mucus-exposed and mucus-free biofilms, 96-well microplates were loaded every 24 h in triplicate with 150 µL of mucus-amended and mucus-free inocula of each isolate. Triplicate 150 µL-aliquots of bacteria-free seawater and mucus-amended seawater without bacteria were used as negative controls. Changes in the phenotype and cell viability of biofilms were determined every 24 h for 168 h in three independent experiments using the Cytation-5 reader and the LIVE/DEAD BacLight Bacterial Viability kit (Invitrogen), as described above.

### Statistical analyses

Beta regressions for modeling beta-distributed dependent variables (e.g., rates and proportions) were performed to test how cytotoxicity in SHK-1 cells (dependent variable, expressed as a percentage) varied over time (five time intervals: 0, 6, 12, 24, 48, and 72 hpi) depending on the infecting *P*. *salmonis* isolate (Psal-103 vs. Psal-104) and growth state (biofilm vs. planktonic). Beta regression analysis was performed with the function ‘betareg’ from the betareg R package^36^. An orthogonal model was built to estimate the effect of each factor on cytotoxicity, as based on LDH production by *P*. *salmonis*-infected SHK-1 cells. A posteriori Tukey’s test was performed using the ‘emmeans’ R package^37^ to identify significant differences (*P* < 0.05) between treatment combinations.

A generalized linear model was used by assuming a lognormal distribution with an identity function to test if three response variables (i.e., SBF index, fluorescence signal of live bacteria in biofilms, and fluorescence ratio of live-to-dead bacteria in biofilms) were affected by the factors of the isolate (Psal-103 vs. Psal-104), suspension media (i.e., nutrient-enriched AUSTRAL medium vs. nutrient-poor seawater or mucus-free vs. mucus-amended seawater), and incubation time (i.e., between 24–480 h [SBF index], 24–360 h [fluorescence signal of live bacteria and the fluorescence ratio of live-to-dead bacteria], and 24–168 h [fluorescence ratio in mucus-amended experiments]). An orthogonal factor model with the three factors was implemented. The lognormal distribution was assumed by the Akaike information criterion using the ‘propagate’ R package^38^. Tukey’s test was performed using the ‘emmeans’ R package^37^ to identify significant differences (*P* < 0.05) between treatment combinations.

Finally, the Gompertz model of four parameters^39^ was fitted to the fluorescence signal of live bacteria in biofilms to predict temporal development. The model is described as $N(t)=N0+C\times exp(-exp(-B\times (t-M)))$, where N(t) is the fluorescence of live bacteria in biofilms at a given time; N0 is the lower asymptote; C is the difference between the lower asymptote and upper asymptote; B is the maximum specific growth rate (μ_max_); t is a given time; and M is the time when B occurs. All analyses were performed using the open-source program R^40^.

## Results

### Cytotoxic effect of biofilm-harvested and planktonic *P*. *salmonis* on SHK-1 cells

**Figure 1:**
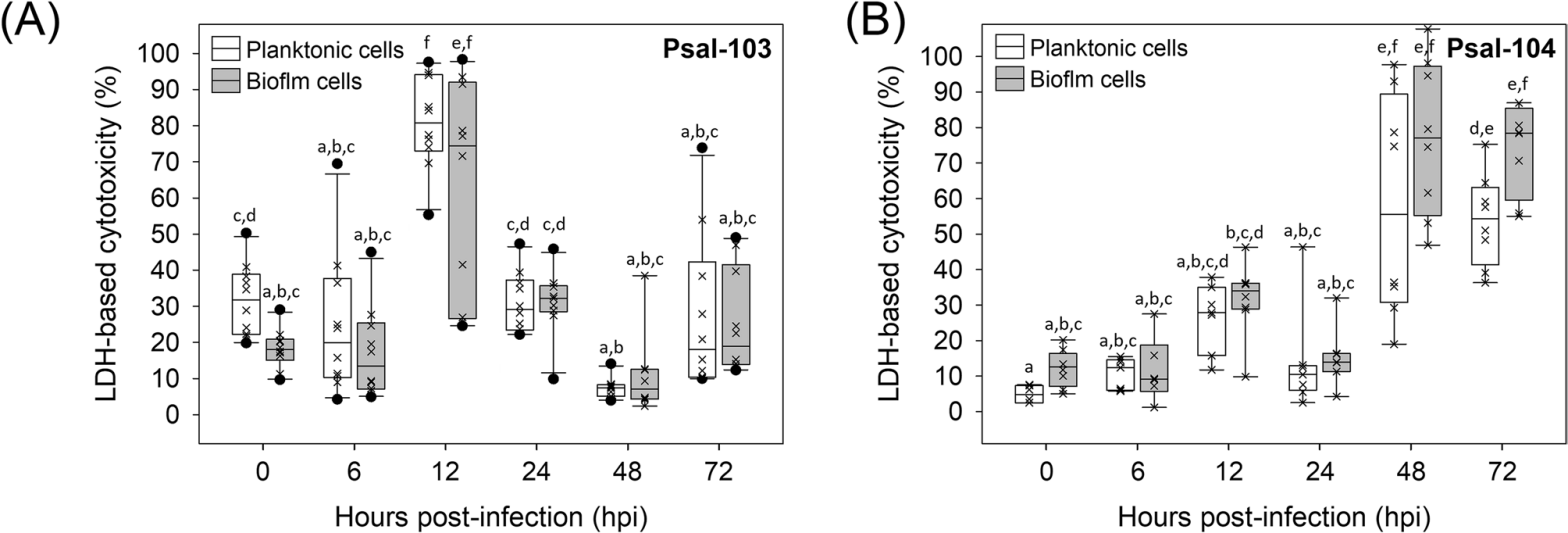
Cytotoxic response of salmon head kidney (SHK-1) cells to infections caused by 288-h old *P*. *salmonis* inocula. Box plots show the cytotoxic response (based on lactate dehydrogenase [LDH] production) of SHK-1 cells to planktonic and biofilm inocula of (**A**) Psal-103 and (**B**) Psal-104 isolates. Each box plot shows the median of data sets ( ×), upper/lower quartiles (boxes), range of percentages (vertical lines), and extreme values (black circles). Different letters denote statistically significant differences (*P* < 0.05) among data sets based on Tukey’s test for treatment combinations. Two or more boxes sharing the same letter mean that no statistically significant differences existed. LDH-based data are representative of three independent experiments.

Interactions of the isolate with growth state (biofilm vs. planktonic) (F-ratio = 12.37, ***P < 0.001) or with hpi (F-ratio = 71.20, ***P = 0.001) significantly affected the general patterns of cytotoxicity induced by *P*. *salmonis* in SHK-1 cells (Table 1). These two interactions determined a cytotoxicity pattern specifically induced by each isolate over time (Fig. 1A,B). Regardless of the source of the *P*. *salmonis* inoculum (biofilm or plankton), isolate Psal-103 was more cytotoxic to SHK-1 cells within the first 12 hpi, with clear significant differences between the cytotoxic effects of both strains at 12 hpi (Fig. 1A,B) (Supplementary Fig. S1). In contrast, isolate Psal-104 was significantly more cytotoxic towards the end of the incubation period, between 48 and 72 hpi (Fig. 1A,B) (Supplementary Fig. S2). The growth state was not a meaningful force for the observed trends in cytotoxicity (F-ratio = 0.51, *P* = 0.475) (Table 1). There were no statistically significant intraisolate differences in cytotoxicity between biofilm-derived and planktonic *P*. *salmonis* at any hpi (Fig. 1A,B).

**Table 1:** Beta regression analysis to model variations in the cytotoxic response of SHK-1 cells to *P*. *salmonis* depending on the infecting isolate, the elapsed time after infection, and the source of the *P*. *salmonis* inoculum (i.e., biofilm vs. planktonic or growth states). The statistical significances of predictors of the beta regression model are denoted as follows: ‘****’*P* ≤ 0.0001; ‘***’*P* ≤ 0.001; ‘**’*P* ≤ 0.01; ‘*’*P* ≤ 0.05, and non-significant (ns) *P* > 0.05. Hours post-infection (hpi); degrees of freedom (df); F-ratio (values of ~ 1 indicate that the predictor does not affect the residual variance).

Model term	df	F-ratio	*P* value
Isolate	1	1.331	0.249^ ns^
hpi	5	45.530	0.001***
Growth state	1	0.509	0.475^ ns^
Isolate × hpi	5	71.204	0.001***
Isolate × growth state	1	12.368	0.000***
hpi × growth state	5	1.631	0.148^ ns^
Isolate × hpi × growth state	5	0.706	0.619^ ns^

### Specific biofilm formation (SBF) indexes

**Figure 2:**
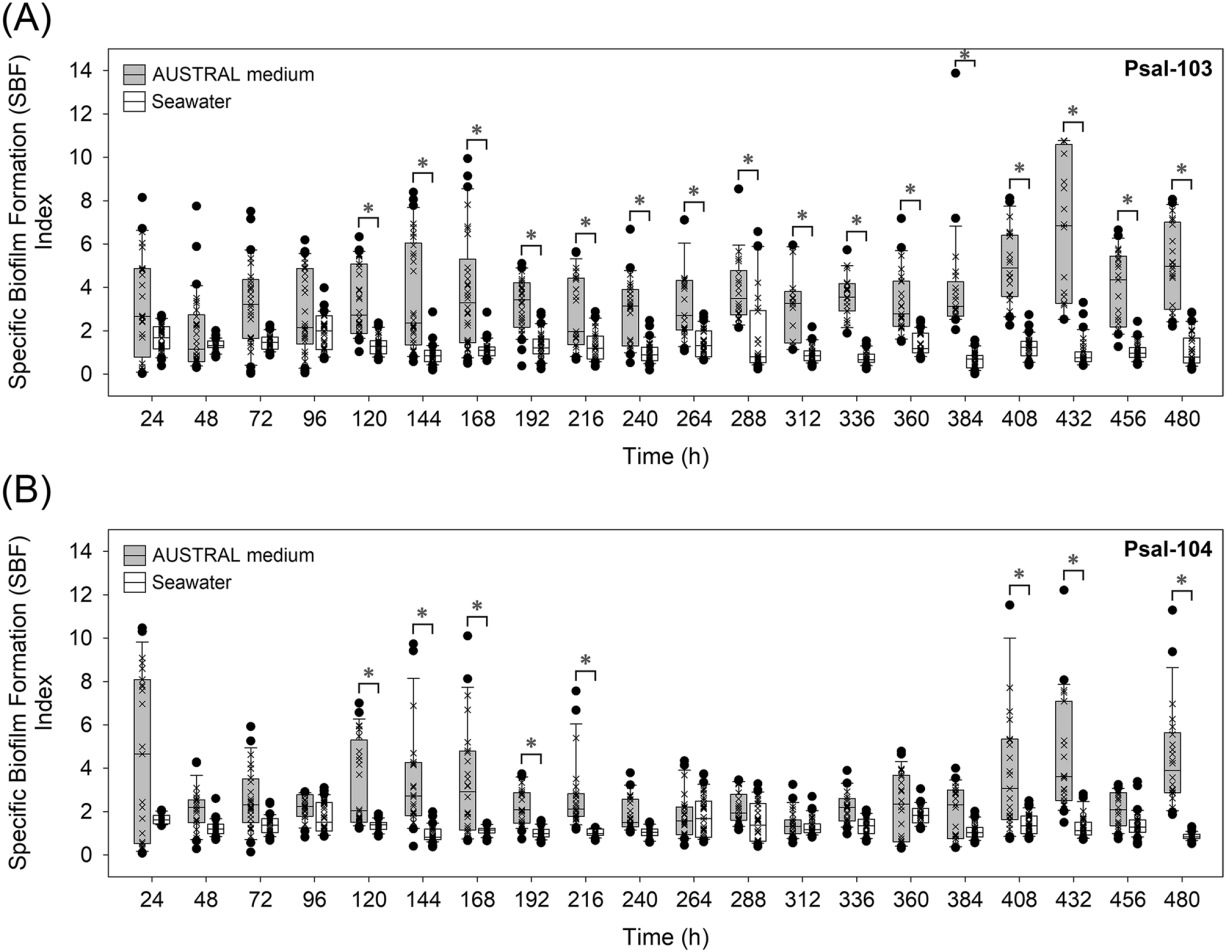
Specific biofilm formation (SBF) indexes. Temporal variations in the SBF index for biofilms formed by *P*. *salmonis* (**A**) Psal-103 and (**B**) Psal-104 in both the nutrient-enriched AUSTRAL medium and the nutrient-poor seawater. Box plots show the medians, upper/lower quartiles (boxes), value ranges (vertical lines), and extreme values (circles) of data sets ( ×). Statistically significant differences for treatment combinations were identified using Tukey’s test. Specifically, the figure shows statistically significant differences (*P* < 0.05) (blue asterisks) in SBF indexes of a given isolate between both suspension media (indicated by brackets) at a specific time. The statistical significances of other treatment combinations are reported in Supplementary Table S1. Data sets are representative of three independent experiments.

The three-factor interaction between isolate, time, and suspension media (seawater vs. AUSTRAL medium) had a highly significant effect on the variability of SBF indexes (Wald = 83.73, ****P < 0.0001) (Table 2). The two isolates showed higher SBF indexes in nutrient-enriched conditions than in seawater, indicating that the nutritional content of the suspension medium had a high statistically significant modulatory effect (Wald = 1,165.92, ****P < 0.0001) (Table 2). Furthermore, the two isolates showed an increasing temporal tendency to form biofilms in the nutrient-enriched medium, especially isolate Psal-103 (Fig. 2A,B). This indicates that variations in the SBF index over time were dependent on the isolate (Wald = 55.67, ****P < 0.0001) (Table 2) in this medium. However, in seawater the two isolates showed a slightly decreasing tendency to form biofilms over time, with decreasing changes being slightly more pronounced for isolate Psal-103 (Fig. 2A,B).

**Table 2:** Generalized linear model results for the effects of growth conditions (i.e., time and suspension media) and isolate on the response variables specific biofilm formation (SBF) index, fluorescence signal of live bacteria in biofilms, and fluorescence ratio of live-to-dead bacteria in biofilms. The statistical significances of the effects are denoted as follows: ‘****’*P* ≤ 0.0001; ‘***’*P* ≤ 0.001; ‘**’*P* ≤ 0.01; ‘*’*P* ≤ 0.05, and non-significant (ns) *P* > 0.05. Degrees of freedom (df). Wald test values closer to zero suggest that the tested effect could be removed from the model.

Effect	df	Wald statistic	*P* value
**SBF index**			
(Intercept)	1	6,251.759	0.000****
Isolate	1	55.671	0.000****
Time	19	227.460	0.000****
Suspension media^a^	1	1,165.919	0.000****
Isolate × time	19	46.727	0.000***
Isolate × suspension media^a^	1	78.773	0.000****
Time × suspension media^a^	19	287.206	0.000****
Isolate × time × suspension media^a^	19	83.726	0.000****
**Fluorescence signal of live bacteria in biofilms**			
(Intercept)	1	1806.946	0.000****
Isolate	1	175.003	0.000****
Time	14	832.884	0.000****
Suspension media^a^	1	19.218	0.000****
Isolate × time	14	86.234	0.000****
Isolate × suspension media^a^	1	10.994	0.000***
Time × suspension media^a^	14	33.668	0.002**
Isolate × time × suspension media^a^	14	11.842	0.619^ ns^
**Fluorescence ratio of live-to-dead bacteria in biofilms**			
(Intercept)	1	6,017.165	0.000****
Isolate	1	15.030	0.000***
Time	14	173.768	0.000****
Suspension media^a^	1	6.006	0.014*
Isolate × time	14	24.481	0.040*
Isolate × suspension media^a^	1	8.068	0.004**
Time × suspension media^a^	14	24.696	0.037*
Isolate × time × suspension media^a^	14	34.520	0.001**
**Fluorescent ratio of live-to-dead bacteria in biofilms: experiments on the effect of mucus on this variable**			
(Intercept)	1	5,187.047	0.000****
Isolate	1	0.178	0.673^ ns^
Time	6	176.526	0.000****
Suspension media^b^	1	240.521	0.000****
Isolate × time	6	9.542	0.145^ ns^
Isolate × suspension media^b^	1	0.443	0.505^ ns^
Time × suspension media^b^	6	32.268	0.000****
Isolate × time × suspension media^b^	6	23.012	0.000**

^a^Seawater or AUSTRAL medium.

^b^Mucus-free or mucus-amended seawater.

In general, no significant differences in the SBF index were detected between Psal-103 and Psal-104 biofilms that had the same development time and that were formed in the same suspension medium (Fig. 2A,B) (Supplementary Table S1). Moreover, isolate Psal-103 showed significant differences in the SBF index between suspension media from 120 h onwards (Fig. 2A) (Supplementary Table S1). Isolate Psal-104 showed such differences at only specific times of biofilm formation (i.e., 408, 432, and 480 h) and from 120 to 216 h (Fig. 2B) (Supplementary Table S1).

### Fluorescence signal of live bacteria in biofilms and the prediction of biofilm formation

**Figure 3:**
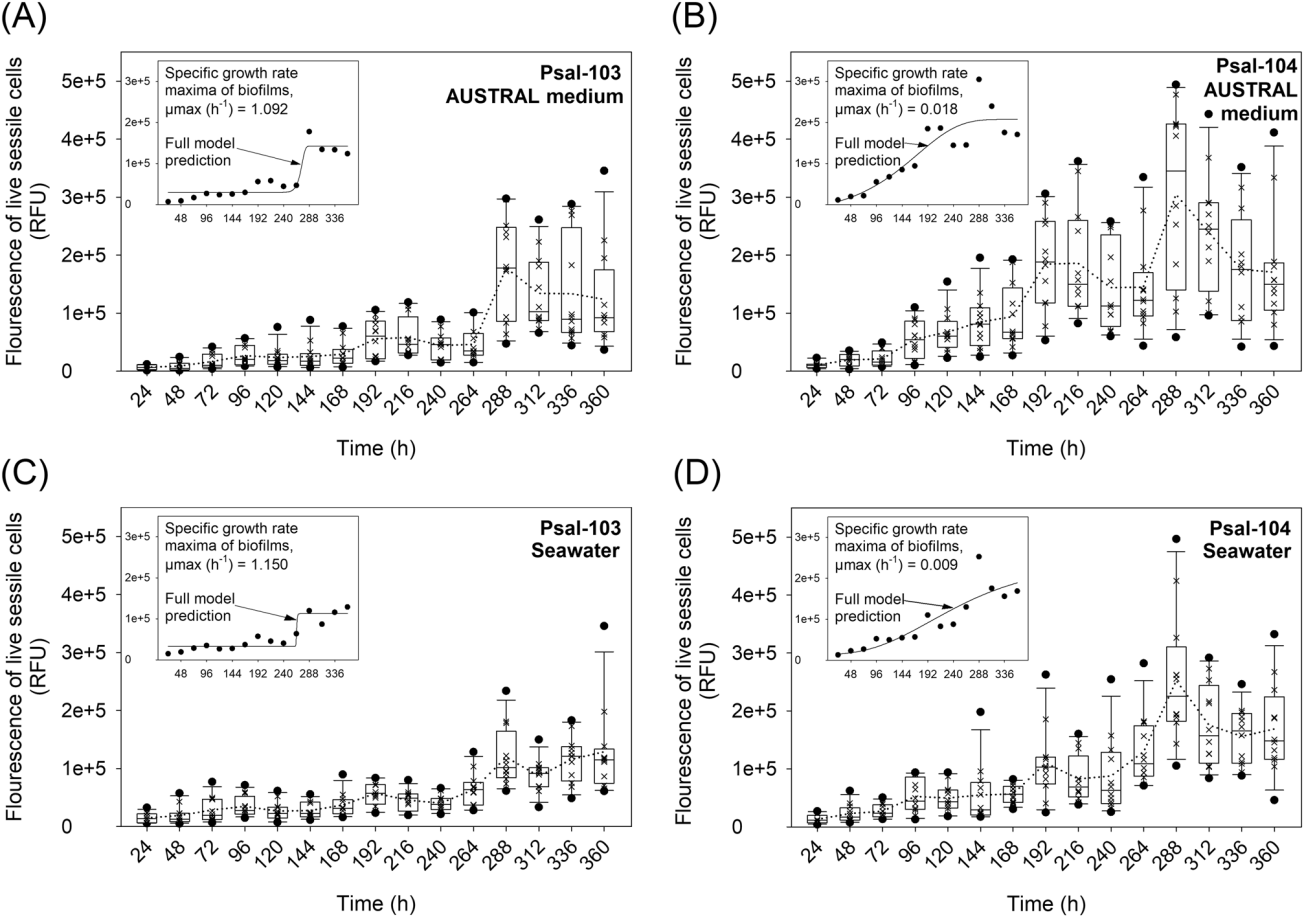
Fluorescence signal of live bacteria in biofilms and sessile growth prediction. Box plots show the increase of live bacteria in biofilms formed by *P*. *salmonis* Psal-103 and Psal-104 over time. This increase was observed in both (**A**, **B**) the nutrient-enriched AUSTRAL medium and (**C**, **D**) the nutrient-poor seawater. Each box plot shows the median of data sets ( ×), upper/lower quartiles (boxes), value ranges (vertical lines), extreme values (circles), and general trends (dotted lines). The statistical significance of treatment combinations (based on Tukey’s test) is reported in Supplementary Table S2. Biofilm growth was predicted by fitting the four-parameter Gompertz model to the fluorescence data of live bacteria (see inner panels). The proportion of variance explained by this model accounted for 84 ± 5.6% (mean ± SD) of variation. Data sets are representative of three independent experiments.

Interactions of suspension media (seawater vs. AUSTRAL medium) with time (Wald = 33.67, **P < 0.01) or with the isolate (Wald = 10.99, ***P < 0.001) significantly affected general patterns of the fluorescence signal of live bacteria in *P*. *salmonis* biofilms (Table 2). Live bacteria tended to be more abundant inside Psal-104 biofilms formed in the AUSTRAL medium than in Psal-103 biofilms formed in the AUSTRAL medium or seawater (Fig. 3A–C), with significant differences at some time points (Supplementary Table S2). For instance, in biofilms formed in the AUSTRAL medium at 48 h and between 120 and 264 h (Fig. 3A,B), the fluorescence signal of live Psal-104 bacteria was significantly higher than that for live Psal-103 bacteria (Supplementary Table S2). Moreover, the fluorescence signal of live bacteria in Psal-104 biofilms formed in the AUSTRAL medium was higher than that emitted by Psal-103 biofilms formed in seawater at 144 h and between 192 and 240 h (Fig. 3B,C) (Supplementary Table S2). There were no statistically significant differences in the fluorescence signal of live bacteria between Psal-103 and Psal-104 biofilms at the same development time when formed in seawater (Fig. 3C,D) (Supplementary Table S2). Likewise, no statistically significant differences in the fluorescence signal of live bacteria were found between biofilms with the same development time formed by the same isolate in different suspension media (Fig. 3A–D) (Supplementary Table S2).

The two isolates formed biofilms that contained live bacteria that tended to significantly increase over time (Supplementary Fig. S3 and S4); the fluorescence signal of live bacteria in biofilms was highly significantly modulated by isolate (Wald = 175, ****P < 0.0001) (Table 2). In general, all *P*. *salmonis* biofilms contained larger and more defined bacteria in the nutrient-enriched medium than in seawater (Supplementary Fig. S3 and S4)). The lag time for biofilm formation was longer in isolate Psal-103 (ca. 260 h) than in isolate Psal-104 (ca. 70–90 h) (inner panels in Fig. 3A–D). While all biofilms reached maximum and comparable levels of a fluorescence signal emitted by live bacteria from 288 h onwards, signifying mature biofilm formation (Fig. 3A–D), Psal-103 biofilms exhibited the highest maximum specific growth rates (μmax) regardless of suspension media (inner panels in Fig. 3A–D). Accordingly, the isolate Psal-103 showed a sudden increase in biofilm formation in a noticeably shorter period of time.

### Variability in the fluorescence ratio of live-to-dead bacteria in biofilms

**Figure 4:**
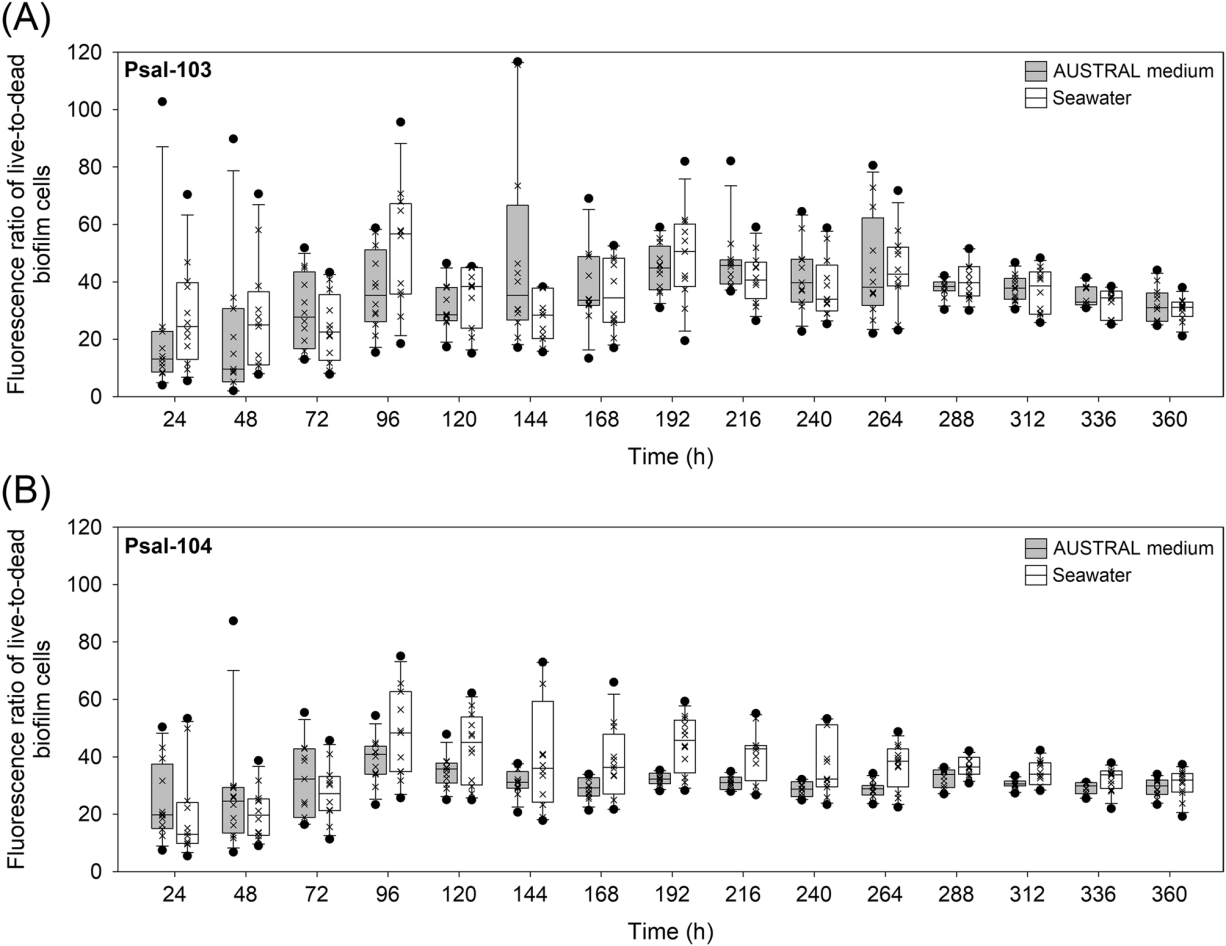
Fluorescence ratio of live-to-dead bacteria in biofilms. Temporal variations in fluorescence ratios for biofilms formed by *P*. *salmonis* (**A**) Psal-103 and (**B**) Psal-104 in both the nutrient-enriched AUSTRAL medium and the nutrient-poor seawater. Box plots show the medians of data sets ( ×), upper/lower quartiles (boxes), value ranges (vertical lines), and extreme values (circles). The statistical significance of treatment combinations (based on Tukey’s test) is reported in Supplementary Table S3. Data were collected from three independent experiments.

The three-factor interaction between isolate, time, and suspension media (seawater vs. AUSTRAL medium) significantly affected the variability in fluorescence ratios of live-to-dead bacteria in biofilms (Wald = 34.52, **P < 0.01) (Table 2). Time was a highly meaningful factor (Wald = 173.77, ****P < 0.0001) (Table 2) for the observed general increase in these ratios (Fig. 4A,B). Isolate Psal-104 tended to show wider fluctuations in viability ratios during earlier biofilm development, whereas isolate Psal-103 showed such fluctuations between the early to middle incubation time points (Fig. 4A,B). This latter result was coupled with a greater lag time in biofilm formation by isolate Psal-103 (as described above). The two isolates showed less variable fluorescence ratios in biofilms from 288 h onwards (Fig. 4A,B); these ratios sometimes were significantly higher than fluorescence ratios determined in the first 48 h (e.g., see Psal-103 and Psal-104 biofilms in AUSTRAL and seawater media, respectively) (Fig. 4A,B) (Supplementary Table S3). Moreover, from 96 h, Psal-104 biofilms in nutrient-poor seawater tended to show higher fluorescence ratios than Psal-104 biofilms in the AUSTRAL medium (Fig. 4B), but this trend was not statistically significant (Supplementary Table S3). No significant differences in fluorescence ratios were found between Psal-103 and Psal-104 biofilms formed in the same suspension medium at the same development time (Fig. 4A,B) (Supplementary Table S3).

### Effect of Atlantic salmon skin mucus on the fluorescence ratio of live-to-dead bacteria

**Figure 5:**
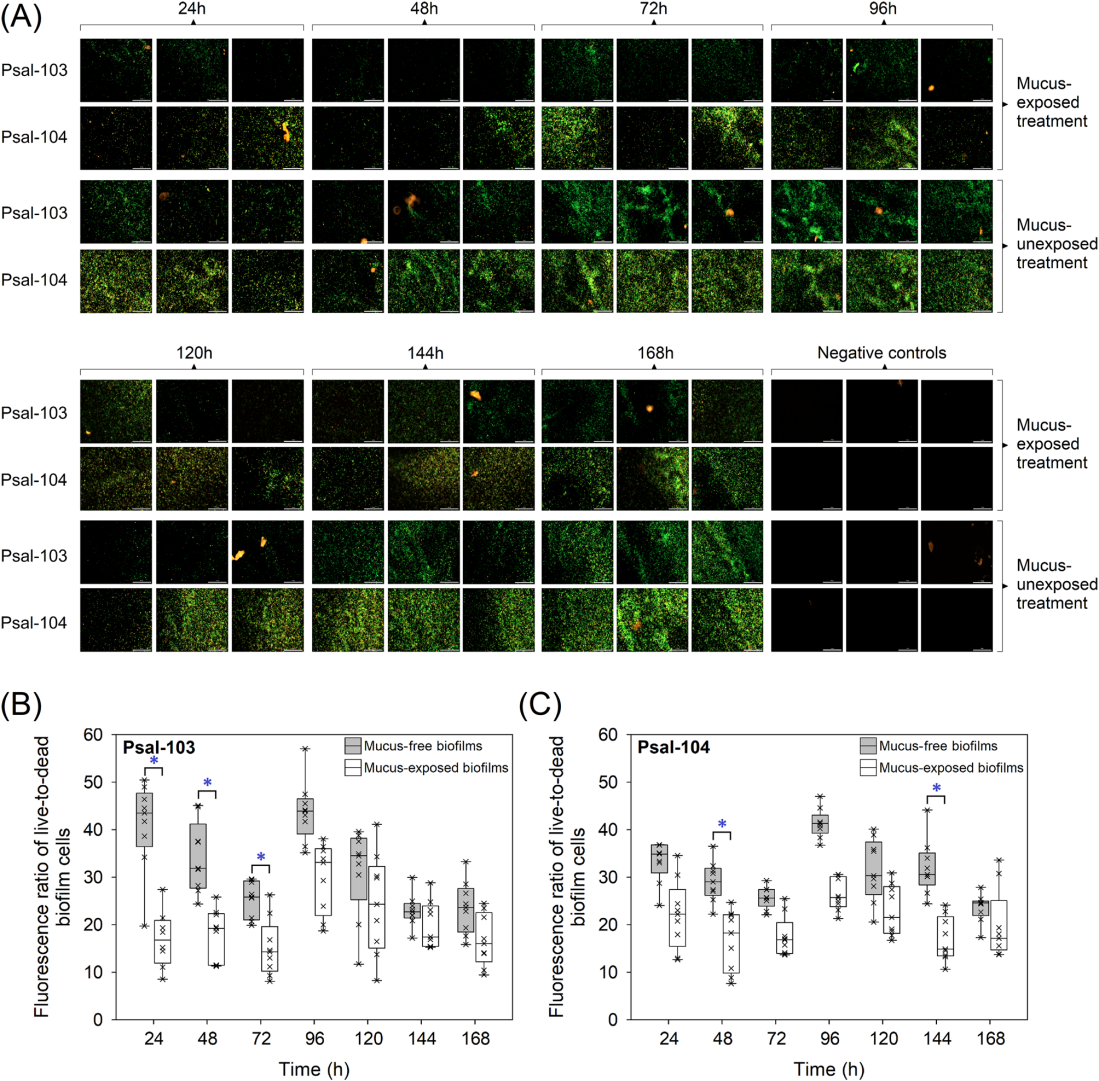
Effect of salmon skin mucus on *P. salmonis* biofilms. (**A**) Automated imaging of biofilms formed in seawater on 96-well microplates. All wells were stained with the LIVE/DEAD Bacterial Viability Kit. Each image contains a white bar scale (100 μm) in the lower right corner. All images were captured from independent wells and are representative of three independent experiments. Fluorescence ratios of live-to-dead bacteria in mucus-free and mucus-exposed biofilms formed in seawater by *P*. *salmonis* (**B**) Psal-103 and (**C**) Psal-104. Box plots show the medians of data sets ( ×), upper/lower quartiles (boxes), value ranges (vertical lines), and extreme values (circles). Data were collected from three independent experiments. Specifically, statistically significant differences (*P* < 0.05) between mucus-free and mucus-exposed biofilms are indicated by blue asterisks and brackets based on Tukey’s test for treatment combinations. The statistical significances of other treatment combinations are reported in Supplementary Table S4.

This effect was tested using skin mucus solutions prepared in seawater to a concentration of 100 µg total protein mL^−1^ (Supplementary Fig. S5). Mucus-amended seawater, with mucus as the potentially exclusive source of carbon and energy, was unable to support the growth of the bacterium under liquid and solid culture conditions (data not shown). These preliminary tests allowed for discarding any noise derived from mucus-stimulated biofilm growth that could have affected tolerance experiments (Fig. 5A–C).

There was a significant effect of the interaction between isolate, suspension media (mucus-free vs. mucus-amended seawater), and time on the variability of the fluorescence ratio of live-to-dead bacteria in biofilms (Wald = 23.01, *P* < 0.05) (Table 2). The two isolates showed similar general patterns of temporal variability in fluorescence ratios of biofilms; however, the magnitude of change between mucus-amended and mucus-free seawater was distinct between isolates, especially during the earlier incubation time points (Fig. 5A–C). The fluorescence ratio of live-to-dead bacteria significantly dropped in mucus-exposed Psal-103 biofilms compared with mucus-free Psal-103 biofilms within the first 72 h (Fig. 5A,B) (Supplementary Table S4). In contrast, mucus-exposed Psal-104 biofilms showed fluorescence ratios significantly lower than mucus-free Psal-104 biofilms only twice over the incubation period (i.e., at 48 h and 144 h) (Fig. 5A,C) (Supplementary Table S4). There were no significant differences in fluorescence ratios between mucus-exposed Psal-103 and Psal-104 biofilms with the same age of development (Supplementary Table S4). However, there were some significant interisolate differences in fluorescence ratios when comparing mucus-exposed biofilms of different development times. For instance, fluorescence ratios of mucus-exposed Psal-103 biofilms at 96 h were significantly higher than those of mucus-exposed Psal-104 biofilms at 48, 72, and 144 h of development (Supplementary Table S4).

## Discussion

Regarding biological levels of organization, biofilms transcend unicellular entities and are widely exploited by bacteria and archaea to face adverse environmental conditions in major habitats on earth^9^. Moreover, biofilms act as virulence determinants that predispose a range of hosts to many bacterial infections^41^. Therefore, the involvement of biofilms in piscirickettsiosis outbreaks cannot be discarded^17^. This could occur through the direct formation of biofilms or biofilm-like aggregates of *P*. *salmonis* on the fish skin^42^, fish eggs^13^, other fish components, and/or indirectly as an environmental seed bank^43^ of the bacterium outside of hosts.

Displays by surface-attached bacteria can range from increased virulence^44^ to biofilm-induced attenuation^45^. In the present study, cytotoxic trials using SHK-1 cells and *P*. *salmonis* derived from 288-h old biofilms (as single bacterial cells and/or aggregates) did not support the attenuation hypothesis. Interactions of the isolate with growth state (i.e., biofilm vs. plankton) (F-ratio = 12.37, ***P < 0.001) or with time (i.e., hpi) (F-ratio = 71.20, ***P = 0.001) significantly affected the general patterns of cytotoxicity induced by *P*. *salmonis* on SHK-1 cells (Table 1). This joint effect could partially be explained by varying quantities of dormant bacteria in the initial inocula used in cytotoxic trials; the two states of *P*. *salmonis* Psal-104 were unculturable on agar plates at the moment of inoculating the SHK-1 cells. This fact could have delayed the cytotoxic effect of isolate Psal-104 on the cell line as compared to planktonic and biofilm Psal-103 inocula (Fig. 1A,B). Biofilm inocula enriched with dormant bacteria have previously been associated with immune-system evasion and lower stimulatory effects on macrophages^46^. No significant intraisolate differences in *P*. *salmonis*-induced cytotoxicity were detected between the biofilm-harvested and planktonic bacteria (Fig. 1A,B). This result should not be extrapolated to in vivo infection cases of salmonids or other bacterial species. For instance, another study that used *Flavobacterium psychrophilum* and the CHSE-214 cell line demonstrated higher virulence in the biofilm state than in the planktonic state^47^. However, the aforementioned lack of differences in *P*. *salmonis*-induced cytotoxicity between biofilm-derived and planktonic bacteria has also been found when infecting the CHSE-214 cell line with *P*. *salmonis* LF-89^ T^ and CA5 (results from another yet-unpublished study by our team). These results suggest that there is a trend towards similarity between biofilm-derived and planktonic *P*. *salmonis* regarding their corresponding cytotoxic effects on fish cell lines.

Freshly dispersed bacteria from mature biofilms can represent a transient, highly virulent lifestyle distinct from true planktonic and biofilm lifestyles^48^. Once detached, these phenotypes could use some of the different entry portals in fish that have been described for *P*. *salmonis*, including the skin and gills in rainbow trout (*Oncorhynchus mykiss*)^49^. *P*. *salmonis* can survive outside of fish for a long time as a free-living bacterium^6^, and transmission may occur in the absence of a vector^50^. This survival behavior could be related to environmental biofilms acting as seed banks for spreading new virulent phenotypes and/or virulent structures of *P*. *salmonis*, such as membrane vesicles^51^. Membrane vesicles play a key role in the biofilm life cycle of some bacterial species^52^, and this may also apply to *P*. *salmonis* biofilms. Linking piscirickettsiosis prevalence to new biofilm-driven scenarios of infection in aquaculture settings requires further investigation. Prior research has observed that acute *P*. *salmonis* infections often generate mortality without gross external clinical signs, while chronically infected fish often show clinical signs such as skin darkness, lethargy, and lateral skin ulcerates, among others^53^. It is currently unknown if *P*. *salmonis* biofilms or dispersed bacteria from these structures (as single-cell entities and/or aggregates) are involved in acute and/or chronic forms of the disease. However, considering the capacity of *P*. *salmonis* to form microcolony-like aggregations in tissues (e.g., in the skeletal muscle of rainbow trout^42^), the participation of biofilm-related structures (e.g., the piscirickettsial attachment complex^13^) during piscirickettsiosis cannot be ruled out, especially in chronic cases, such as with bacterial biofilms involved in other fish pathologies^54^.

Studies on biofilm formation by *P*. *salmonis* are scarce. This bacterium has the potential to form biofilms on living substrates^13,14^ and does form biofilms on non-living substrates^7,15,16^. Only two previous studies have described *P*. *salmonis* biofilms as a strategy of bacterial survival and resistance to external stressors, such as nutritional stress^7,15^. However, both studies used marine broth under the assumption of it being a nutritionally limited medium. In fact, marine broth has been described as a nutrient-rich medium from which several heterotrophic bacteria have been isolated, including bacteria from the genera *Vibrio*, *Pseudomonas*, *Oceanospirillum*, *Aeromonas*, and *Flavobacterium*, among others^55^. Moreover, one of the previous studies used the type strain LF-89^ T^ grown at 23°C^7^. This is at least 5 °C higher than the optimal temperature for the species (i.e., 15–18 °C)^56^, in addition to being an unrealistic temperature for aquaculture settings. In this case, the effect of nutritional stress on the behavior of *P*. *salmonis* biofilm could be biased by stress due to temperature. Since the composition of marine broth is far from the real nutritional conditions for biofilm formation in the field (i.e. in aquaculture settings), the present study used seawater at 18 °C to emulate a natural condition and compared this against the nutrient-enriched AUSTRAL medium at the same temperature. In general, changes in the SBF index are linked to changes in live and dead bacteria, as well as to the biofilm matrix^57^. These three components together form the bulk fraction of the biofilm structure. Currently obtained SBF data showed that Psal-103 and Psal-104 biofilms tolerated a long-term (480 h) drop in nutrients in the AUSTRAL medium. Similarly, biofilms were stable in nutrient-poor seawater over the same period of time (Fig. 2A,B). All biofilms showed higher SBF indexes in the AUSTRAL medium than in seawater, indicating that the nutritional contents of the suspension medium significantly affected the bulk fraction of the biofilm structure over time (Wald = 1,165.92, ****P < 0.0001) (Table 2).

There were no significant changes in the SBF index for a given isolate in a specific medium over time (Fig. 2A,B). These results contrast with the notion that nutrient-depleted conditions lead to cell detachment and biofilm disaggregation^58^. Psal-103 biofilms formed in the AUSTRAL medium and seawater showed significant differences in the SBF index from 120 h onwards, but Psal-104 biofilms showed the same differences only at specific times (Fig. 2A,B). Moreover, non-significant intraisolate differences in the fluorescence of live bacteria were found when comparing both suspension media. These results suggest that there was an isolate-dependent response of *P*. *salmonis* biofilms to nutrient availability, as reflected by the SBF index. Higher nutrient levels are associated with higher exopolymer amounts in the extracellular matrix of environmental biofilms^59,60^. Accordingly, significant differences in the SBF index (but not in the fluorescence of live bacteria) between suspension media, especially in the case of the Psal-103 isolate, could have been linked to differences in exopolymer production between the nutritionally contrasting conditions of suspension media. In addition, changes in the SBF index (Fig. 2A,B) were accompanied by a sustained temporal increase of the live bacterial fraction in all biofilms, even in biofilms formed in seawater (Fig. 3A–D). Nevertheless, all *P*. *salmonis* biofilms appeared to contain larger and more defined bacteria in the nutrient-enriched medium than in seawater (Supplementary Figs. S3 and S4). A similar observation was previously reported in another bacterial model that was also induced to form biofilm under static growth conditions^61^.

Suspension media (i.e., seawater vs. AUSTRAL medium) in association with the isolate had a significant joint effect on the temporal increase of the fluorescence signal of live bacteria in biofilms (Wald = 10.99, ***P < 0.001) (Table 2). This effect was associated with isolate-dependent variations in maximum specific growth rate (μ_max_) and lag time during biofilm formation (Fig. 3A–D). There were no statistically significant differences in the fluorescence of live bacteria between the AUSTRAL medium and seawater for biofilms of the same isolate and incubation time (Supplementary Table S2). Consequently, maximum specific growth rates (μ_max_) and lag times computed for biofilms formed in different suspension media by the same isolate were highly similar. These two parameters were, therefore, relatively insensitive to contrasting nutritional conditions between suspension media (inner panels in Fig. 3A–D). This observation partially aligns with a previous study indicating that different nutrient concentrations can lead to differences in lag-phase duration during biofilm formation (by *Pseudomonas* sp.) but not to significant changes in maximum specific growth rates^62^. Important changes in the lag times and μ_max_ of bacterial biofilms could also be induced by factors such as mechanical shear stress^63^ and assimilable organic carbon availability^64^, respectively. In the present study, interisolate differences in lag time reflected differences that emerged in the early colonization stage (Fig. 3A–D). These interisolate discrepancies could be associated with variations in colonization efficiency on surfaces, which would be mediated by differences in the physiological adaptation of freshly adhered bacteria^65^. The present study reports the first kinetics model of biofilm formation by *P*. *salmonis*, which could be useful to understand the development of biofilm-based infections^66^.

Seawater is a nutritionally depleted medium compared with the AUSTRAL medium. Additionally, some degree of nutrient depletion in both suspension media can be assumed over the course of biofilm formation. However, general conclusions regarding the role of starvation on biofilm development should be made with caution, as biofilm stages that are highly dependent on cell viability, such as detachment, can be both favored or limited by low nutrient loads^67^. *P*. *salmonis* biofilms showed higher fluorescence ratios of live-to-dead bacteria during the entire incubation period (Fig. 4A,B), without significant intraisolate differences between suspension media (Supplementary Table S3). A net detachment stage of bacteria from biofilms conformed mostly by live bacteria was not detected (Supplementary Figs. S3 and S4). This result contrasts with previous findings on biofilms formed by fish pathogens of the genus *Tenacibaculum*, which were characterized by an important contribution of dead bacteria towards the late stages of biofilm development (i.e., at 96 h)^28,68^. The present results also contrast with previous findings in indicating that biofilms are dominated by dead bacteria in nutrient-poor media as compared to biofilms in nutrient-enriched media^69^ or vice versa^61^. The application of the LIVE/DEAD dye on biofilms with high amounts of extracellular nucleic acids can produce a spurious detection of dead bacteria^70^. This artifact would be more critical in mature biofilms due to DNA release from aged biofilms over time (e.g., by cell lysis and/or partial loss of membrane integrity, in addition to the release from living bacteria)^71^. However, fluorescence ratios of live-to-dead bacteria from 288 h onwards were less variable and were comparable with those from the early stages of biofilm formation (Fig. 4A,B). Fluorescence ratios were even sometimes significantly higher than those determined in the first 48 h (e.g., see Psal-103 and Psal-104 biofilms in AUSTRAL and seawater media, respectively) (Supplementary Table S3). It should therefore be noted that a significant detection of false dead bacteria in the late stages of biofilm formation is very unlikely. On the other hand, wider fluctuations in the fluorescence ratios of live-to-dead bacteria between the early and middle incubation times (Fig. 4A,B) were mostly linked to the lag phase of biofilm formation (Fig. 3A–D). These fluctuations can reflect changes in the metabolic adaptation of freshly adhered bacteria, as well as the influence of extracellular nucleic acid-coated colonizers^72,73^ facilitating the initial phases of biofilm formation^74^. However, this latter point requires further research since extracellular nucleic acids could also inhibit biofilm formation in some bacterial species^75^.

Even though antibody levels against *P*. *salmonis* in the fish-mucus samples used in the biofilm experiments were not determined by the present study, these samples were collected from a fish farm with no history of piscirickettsiosis or immunization against its etiological agent, which suggests a generalized lack of antibodies against the bacterium. However, it is important to note that the immunity of salmonids based on antibodies against *P*. *salmonis* does not guarantee the resistance of fish to piscirickettsiosis. Indeed, there are 33 government-approved vaccines against piscirickettsiosis in Chile, but effectiveness is so low^76^ that antibiotics are still the treatment of choice for the industry^17,77^.

Despite the mucosal immunological barrier of fish skin against pathogens^22^, injuries on body surfaces are a typical clinical sign of piscirickettsiosis infection^2^. A free pass to bacterial adhesion-dependent processes, such as colonization and biofilm formation^17,42^, could take place on fish skin if *P*. *salmonis* can override the mucosal barrier. Remarkably, the interaction between fish skin mucus and *P*. *salmonis* is far from being understood, and much less is known about the persistence and infection strategies of this bacterium^49,78^. In the present study, isolate Psal-103 was sensitive to salmon skin mucus during the early stages of biofilm formation in seawater, but mucus tolerance was observed in Psal-103 biofilms from 96 h onwards (Fig. 5A,B). In contrast, Psal-104 biofilms appeared much more tolerant to the salmon skin mucus over time (Fig. 5A,C). Fish skin mucus can stimulate bacterial biofilm development^79^ in a way similar to that previously observed in *Flavobacterium columnare* with catfish mucus (at 20 µg mL^−1^)^80^. Preliminary experiments in the present study indicated that salmon skin mucus was unable to stimulate the growth of *P*. *salmonis*. However, biofilm-forming *P*. *salmonis* did tolerate a high concentration of salmon skin mucus (100 µg mL^−1^), despite epidermal mucus being recognized as a major determinant in fish health^81^. This latter notion is supported by the role of epidermal mucus in the innate immunity of the host^82,83^ and by the antibacterial properties of epidermal mucus^84^, either as crude mucus or as aqueous mucus extracts against bacterial human and fish pathogens^85^. For example, after *Streptococcus phocae* is exposed to skin mucus from salmonids, interstrain differences in growth occur depending on the mucus donor species^86^.

In summary, our results provide the first insights into the in vitro virulence-related capacities of *P*. *salmonis* harvested from biofilms as single bacterial cells and/or aggregates. The two isolates triggered a cytotoxic response in SHK-1 cells, without intraisolate variations between the planktonic and biofilm-harvested bacteria. However, this response could depend on interisolate differences on colonized surfaces. In general, biofilm formation relied on the initial colonization step, which, in turn, determined the length of the lag phase and was associated with wider fluctuations in biofilm viability. Interisolate differences in the lag phase emerged regardless of the nutritional contents of the suspension medium. Furthermore, the two isolates reached a mature biofilm stage from ca. 288 h onwards, when fluctuations in biofilm viability were narrower. All of these interisolate differences in biofilm formation were not reflected by changes in the SBF index. A net detachment stage of bacteria from biofilms was not detectable by SBF indexes, automated imaging, or fluorescent measurements. Diverse biologically active molecules present in the fish skin mucus against diseases, such as those modulating the innate immune response^23^, appeared not to be an obstacle to tolerances showed by *P*. *salmonis*. In fact, in the present study, the mean activity of the lysozyme (a component of the innate immune defense in fish) in skin-mucus samples adjusted to 100 µg total protein mL^−1^ was equal to 74 ± 11 U mL^−1^. Overall, this study supports the notion that *P*. *salmonis* forms viable, stable, and fish skin-mucus tolerant biofilms on abiotic surfaces in aquaculture settings under conditions of severe nutrient starvation. Additionally, the presented results underscore the need for rigorous environmental screening of the major *P*. *salmonis* reservoirs that could be involved in the persistence and transmission of piscirickettsiosis.

## Supplementary information

Supplementary figures.

Supplementary tables.

## Data Availability

The authors declare that the data are available on request due to privacy/ethical restrictions.
