# Supplementary material for: Improved understanding of biofilm development by Piscirickettsia salmonis reveals potential risks for the persistence and dissemination of piscirickettsiosis
Source: Sci Rep. 2020 Jul 22;10:12224. doi: 10.1038/s41598-020-68990-4 (PMC7376020; doi:10.1038/s41598-020-68990-4)
Supplement: Supplementary file 1 — Supplementary figures. [file 41598_2020_68990_MOESM1_ESM.pdf]

Supplementary Information

Improved understanding of biofilm development by *Piscirickettsia salmonis* reveals potential risks for the persistence and dissemination of piscirickettsiosis

Héctor A. Levipan[1]*, Rute Irgang[2, 3], Alejandro Yáñez[3, 4] & Ruben Avendaño-Herrera[2, 3, 5]*

[1]Departamento de Biología, Facultad de Ciencias Naturales y Exactas, Universidad de Playa Ancha, Valparaíso, Chile.

[2]Universidad Andrés Bello, Laboratorio de Patología de Organismos Acuáticos y Biotecnología Acuícola, Facultad de Ciencias de la Vida, Viña del Mar, Chile.

[3]Interdisciplinary Center for Aquaculture Research (INCAR), Universidad Andrés Bello, Viña del Mar, Chile.

[4]Facultad de Ciencias, Universidad Austral de Chile, Valdivia, Chile

[5]Centro de Investigación Marina Quintay (CIMARQ), Universidad Andrés Bello, Quintay, Chile.

Keywords: piscirickettsiosis, cytotoxic *Piscirickettsia salmonis*, biofilm viability, salmon skin mucus, and reservoir

***Correspondence to:**

Héctor A. Levipan: hector.levipan@upla.cl, hlevipan46@gmail.com

Rubén Avendaño-Herrera: reavendano@yahoo.com, ravendano@unab.cl

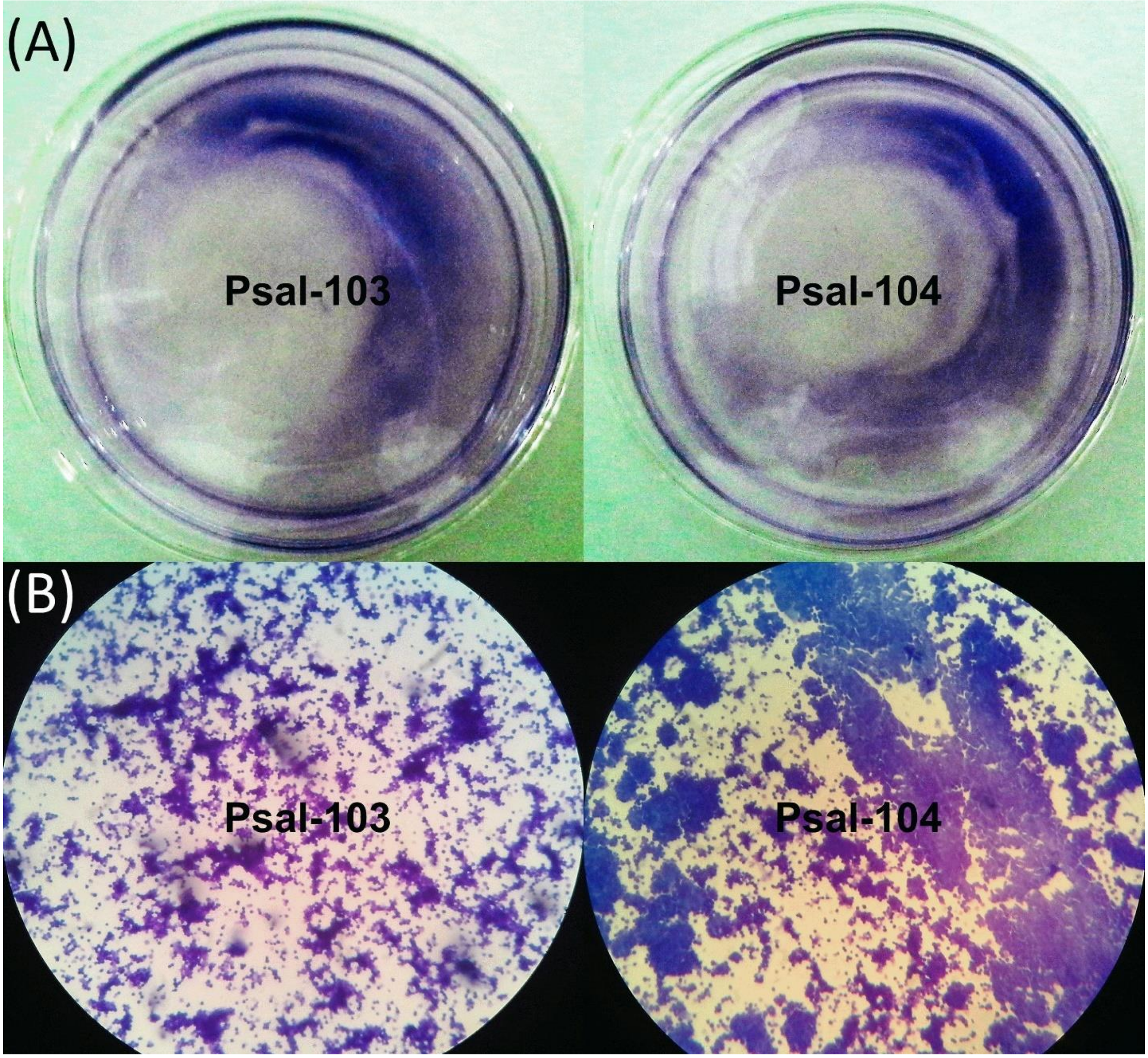

Figure S1. Biofilms formed on glass Petri dishes. (A) Macrograph of CV-stained biofilms formed by *P. salmonis* Psal-103 and Psal-104 after a 288 h incubation period. **(B)** Micrograph of the same CV-stained biofilms shown in **(A)** under 1000X magnification.

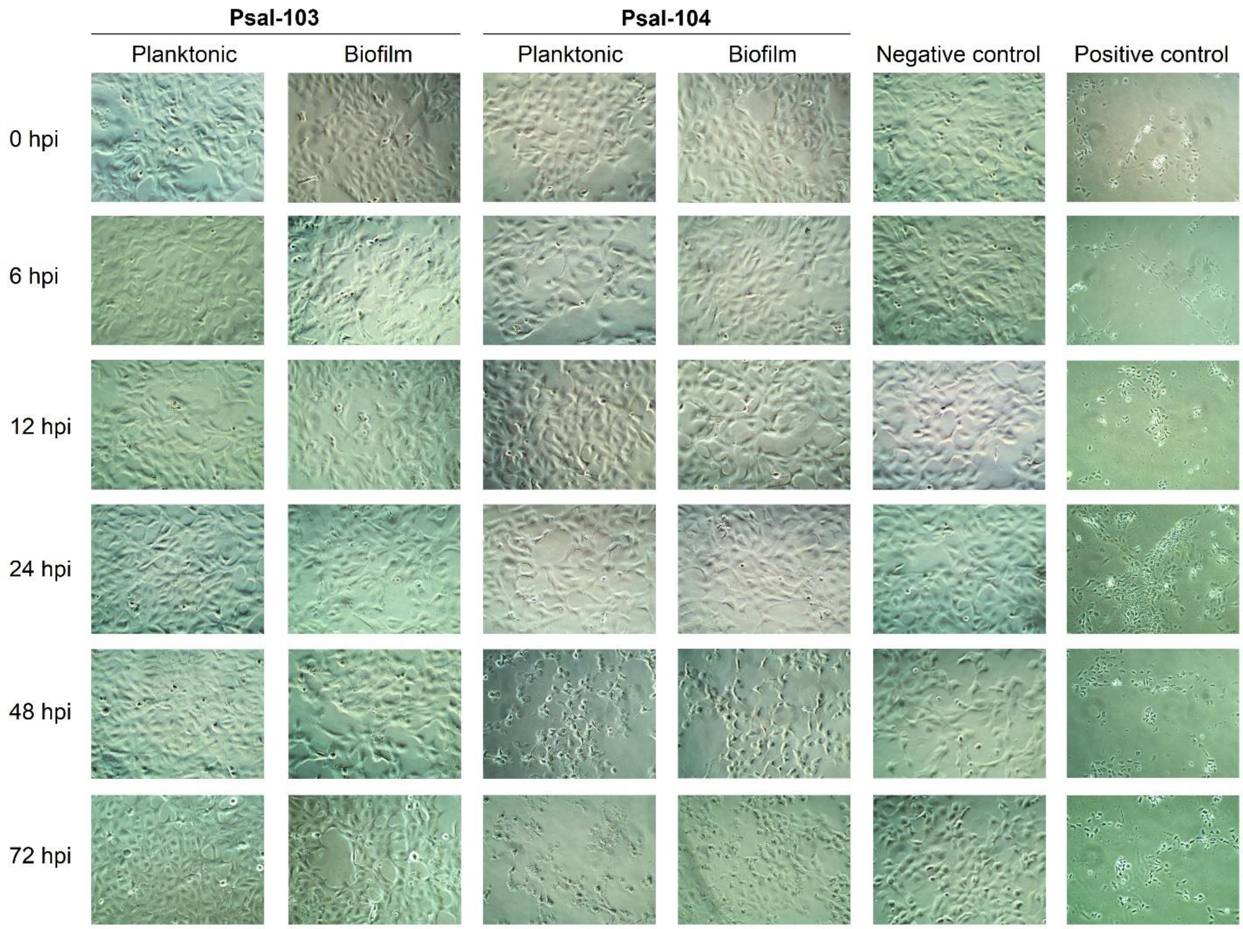

Figure S2. Phenotypic temporal changes in SHK-1 cells infected with 288-h old *P. salmonis* inocula. Bacterially infected SHK-1 cells are compared with negative (i.e., non-infected SHK-1 cells) and positive controls (i.e., SHK-1 cells incubated with 1% Triton X-100). SHK-1 cell micrographs are representative of three independent experiments.

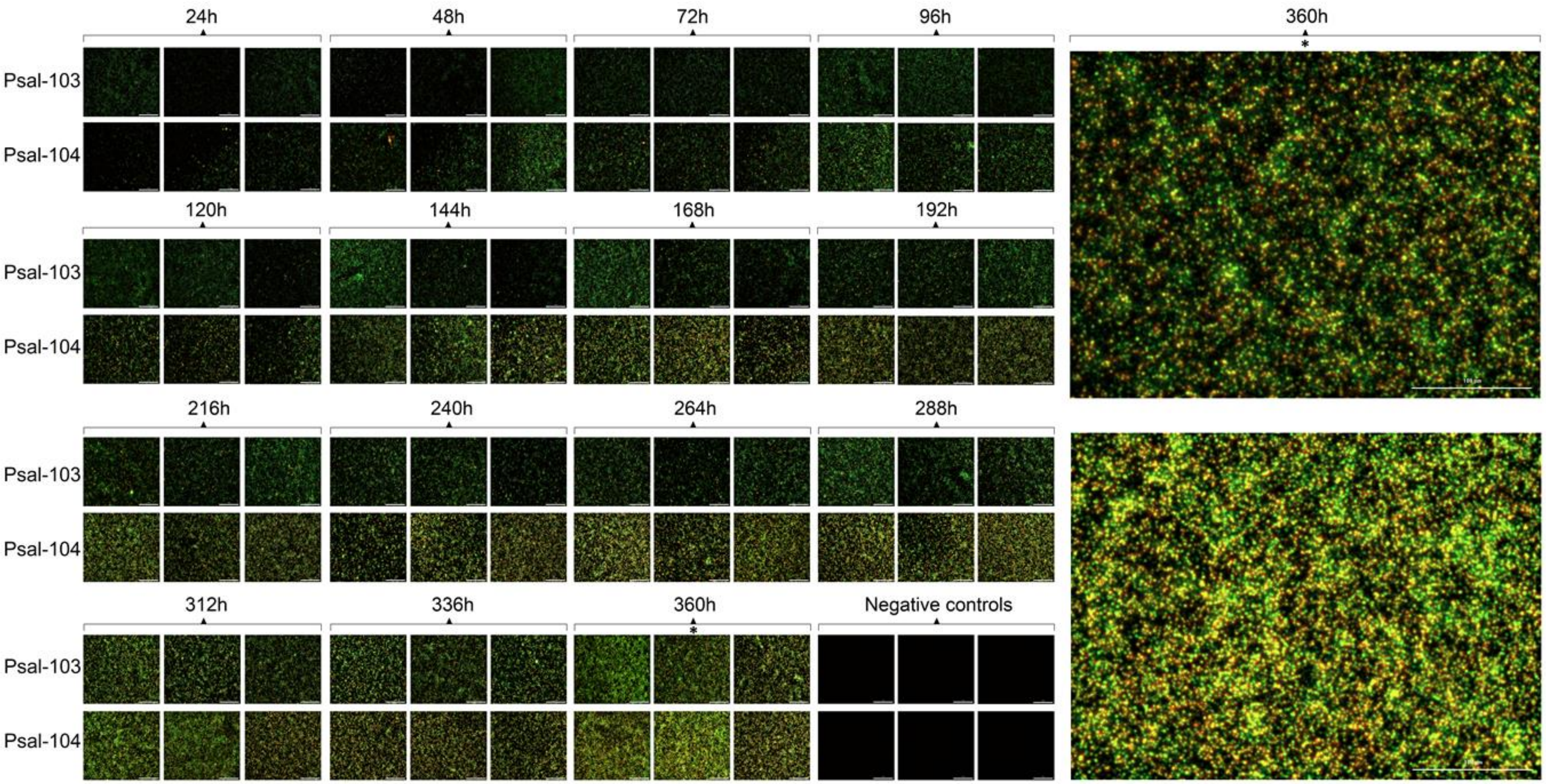

Figure S3. Automated imaging of *P. salmonis* biofilms formed in the nutrient-enriched AUSTRAL medium over time on 96-well microplates. All wells (i.e., with and without biofilms) were stained with the LIVE/DEAD Bacterial Viability Kit. A white scale bar (100 µm) is located in the lower right corner of every image. Zoomed images of 360-h old biofilms show details (asterisk-labeled wells). All images were captured from independent wells and are representative of three independent experiments.

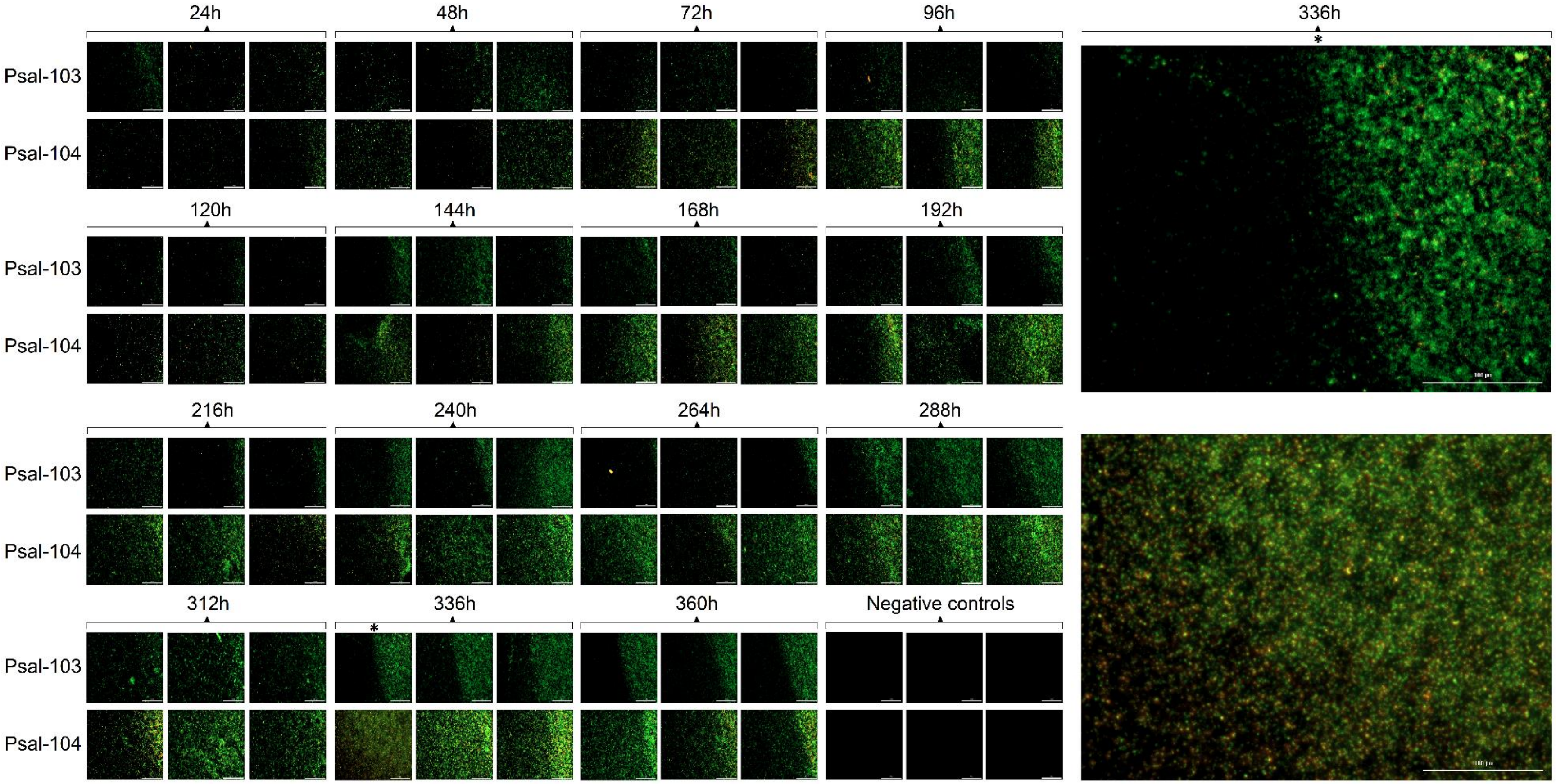

Figure S4. Automated imaging of *P. salmonis* biofilms formed in low-nutrient seawater over time on 96-well microplates. All wells (i.e., with and without biofilms) were stained with the LIVE/DEAD Bacterial Viability Kit. A white scale bar (100 µm) is located in the lower right corner of every image. Zoomed images of 336-h old biofilms show details (asterisk-labeled wells). All images were captured from independent wells and are representative of three independent experiments.

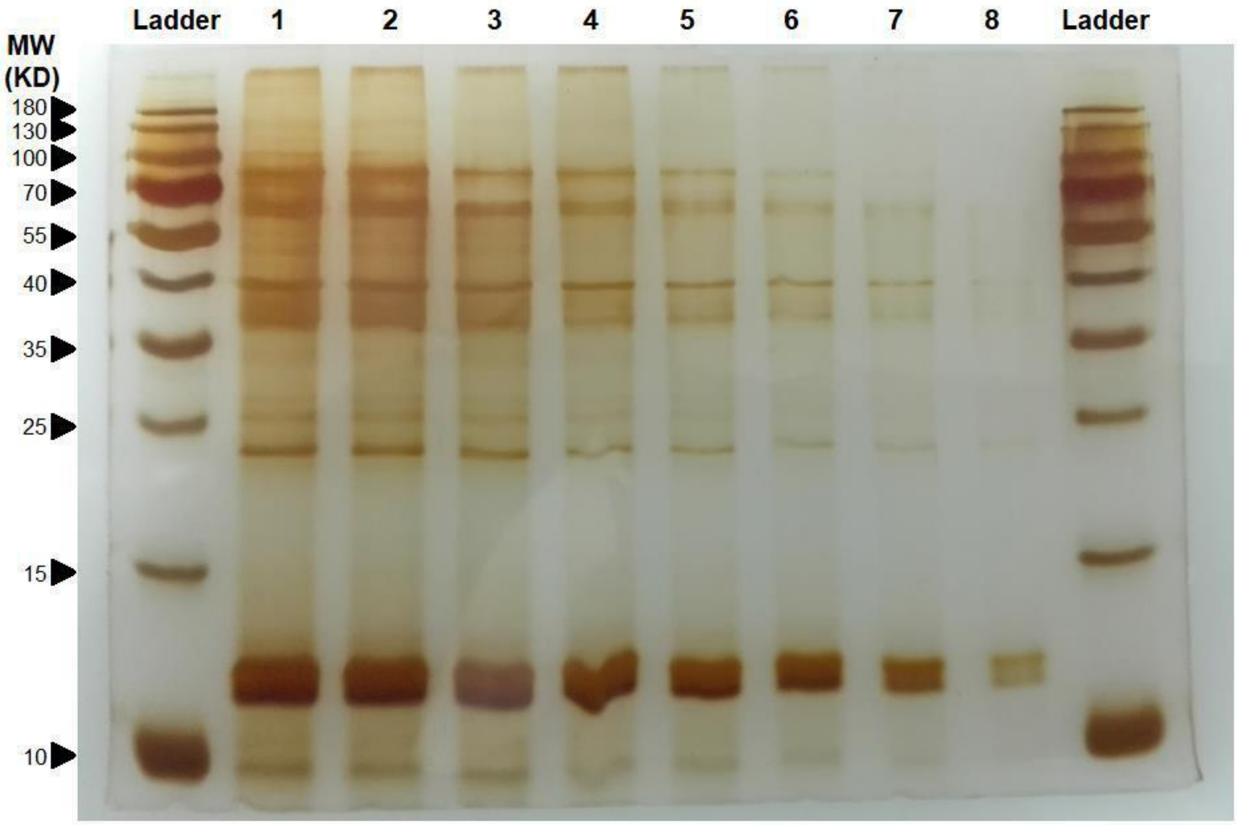

Figure S5. Full-length silver-stained SDS-PAGE gel (12%) for protein profiling of skin-mucus samples from *Salmo salar*. Ladders: 5 µL of the PageRuler Prestained Protein Ladder, range: 10-180 kD (ThermoFisher Scientific). Lines 1-8: 8, 7, 6, 5, 4, 3, 2, and 1 µg of mucosal total protein loaded per line. MW, molecular weight.
